# Screening Marine Microbial Metabolites as Promising Inhibitors of *Borrelia garinii*: A Structural Docking Approach towards Developing Novel Lyme Disease Treatment

**DOI:** 10.1155/2024/9997082

**Published:** 2024-02-29

**Authors:** Zarrin Basharat, Sadia Sattar, Ammar Abdulraheem Bahauddin, Abdulaziz K. Al Mouslem, Ghallab Alotaibi

**Affiliations:** ^1^Alpha Genomics (Private) Limited, Islamabad 45710, Pakistan; ^2^Molecular Virology Labs, Department of Biosciences, COMSATS University Islamabad, Islamabad Campus, Islamabad 45550, Pakistan; ^3^Department of Pharmacology and Toxicology, Taibah University, Madinah Al-Munawarah, Saudi Arabia; ^4^Department of Pharmaceutical Sciences, College of Clinical Pharmacy, King Faisal University, Al Ahsa 31982, Saudi Arabia; ^5^Department of Pharmacology, College of Pharmacy, Al-Dawadmi Campus, Shaqra University, Shaqra, Saudi Arabia

## Abstract

Lyme disease caused by the *Borrelia* species is a growing health concern in many parts of the world. Current treatments for the disease may have side effects, and there is also a need for new therapies that can selectively target the bacteria. Pathogens responsible for Lyme disease include *B. burgdorferi*, *B. afzelii*, and *B. garinii*. In this study, we employed structural docking-based screening to identify potential lead-like inhibitors against the bacterium. We first identified the core essential genome fraction of the bacterium, using 37 strains. Later, we screened a library of lead-like marine microbial metabolites (*n* = 4730) against the arginine deiminase (ADI) protein of *Borrelia garinii*. This protein plays a crucial role in the survival of the bacteria, and inhibiting it can kill the bacterium. The prioritized lead compounds demonstrating favorable binding energies and interactions with the active site of ADI were then evaluated for their drug-like and pharmacokinetic parameters to assess their suitability for development as drugs. Results from molecular dynamics simulation (100 ns) and other scoring parameters suggest that the compound CMNPD18759 (common name: aureobasidin; IUPAC name: 2-[(4*R*,6*R*)-4,6-dihydroxydecanoyl]oxypropan-2-yl (3*S*,5*R*)-3,5-dihydroxydecanoate) holds promise as a potential drug candidate for the treatment of Lyme disease, caused by *B. garinii*. However, further experimental studies are needed to validate the efficacy and safety of this compound *in vivo*.

## 1. Introduction

*Borrelia garinii* belongs to the genus *Borrelia* and is responsible for causing Lyme disease [1, 2]. It is the most common tick-borne disease in the Northern Hemisphere, with over 300,000 new cases reported each year in the United States alone [3]. It is also the common *Borrelia* sp. found in Europe and Asia, where it is transmitted to humans through the bite of infected ticks [4]. This bacterium has a complex life cycle and can survive and persist in different hosts, including the vector tick and humans [5, 6]. After infection, *B. garinii* can cause a range of symptoms associated with Lyme disease, including fever, fatigue, headache, and a characteristic bull's-eye rash [7, 8]. If left untreated, it can spread to other parts of the body and cause symptoms like joint pain, meningitis, and cardiac issues [9, 10]. The diagnosis of Lyme disease entails a comprehensive approach, integrating clinical symptom presentation with confirmation through blood tests and PCR assays [11, 12]. The standard treatment protocol typically includes a course of antibiotics, such as doxycycline or amoxicillin. The prognosis for patients is generally favorable, particularly when the disease is diagnosed early and treated promptly [13]. There is a need for newer drug leads targeting this bacterium, given the limitations associated with the antibiotics currently in use. They may effectively kill the bacterium during the early stages of infection but are often less effective in the later stages of the disease when the bacteria is disseminated to various organs and tissues [14]. In addition, the emergence of antibiotic-resistant strains of *B. garinii* is a growing concern, as this limits the effectiveness of existing antibiotics and poses a significant threat to public health [15, 16].

The discovery of new drugs and targets is critical to overcome this challenge and improve the efficacy and safety of Lyme disease treatment*. B. garinii* has several enzymes that allow it to evade the host immune system and establish persistent infection [17]. These enzymes could be targeted by small molecule inhibitors to prevent survival in the host. Targeting essential genes and proteins that are required for its survival and replication through subtractive proteomics is one strategy. Complementary methods like pan-proteomics are a powerful approach for the narrowing down of new therapeutic targets from the core region of the genome [18]. *In silico* methods, which rely on computational modeling and simulation, have tremendous potential for swift therapeutic target screening and inhibitor design [19, 20]. This approach can be used to screen a large number of potential drug candidates in a relatively short period, in contrast to slow and expensive traditional drug discovery methods. Current methods enable researchers to analyze vast amounts of genomic and structural data rapidly and efficiently. They can be used to identify potential therapeutic targets based on their essentiality, conservation, and druggability. Moreover, computational methods can be used for the design and optimization of small molecule inhibitors capable of selectively and effectively targeting these specific molecular targets [21]. Focusing on conserved proteins across various strains can enhance the efficacy of inhibition and decrease the probability of antibiotic resistance. This approach may involve utilizing natural products or similar drug-like compounds. The application of the pan-proteome concept and combination of these *in silico* techniques to select therapeutic protein targets from *B. garinii* have the potential to identify new targets and improve current therapies for Lyme disease treatment. Furthermore, this approach can contribute to the development of new therapies against the resistant varieties of *B. garinii* strains.

In this study, we selected marine-derived microbial metabolites for screening as they are a rich source of bioactive compounds with potential therapeutic applications, including the development of novel antibiotics [22]. Numerous marine bacterial and fungal species have developed distinctive metabolic pathways, yielding natural products that confer adaptive advantages for their survival in the challenging marine environment [23]. Their metabolites often possess antimicrobial activity against a range of pathogenic bacteria, including antibiotic-resistant strains [24]. One example of a marine organism-sourced bacterial inhibitor is salinosporamide A, which is produced by the bacterium *Salinispora tropica* [25]. This compound is a potent inhibitor of the proteasome, essential for the survival of many pathogenic bacteria, including *Mycobacterium tuberculosis* and *Staphylococcus aureus* [26]. Salinosporamide A has shown promise in preclinical studies as a potential treatment for multiple myelomas as well [27]. Another example of a marine actinomycete-derived MRSA inhibitor is marinopyrrole A [28, 29]. Quorum-sensing inhibitors from marine *Oceanobacillus* sp. [30] and *Staphylococcus hominis* [31] have also been reported in the literature. By disrupting quorum sensing, bacterial colonization and biofilm formation may be prevented in the pathogens. In addition, the efficacy of traditional antibiotics may also be enhanced. Therefore, inhibitors based on marine-derived natural products hold significant promise as novel antibiotics. These have the potential to address antibiotic-resistant infections, thereby enhancing the efficacy and safety of existing treatments. In this study, a marine microbe-derived metabolite library containing lead-like compounds was screened against the arginine deiminase (ADI) enzyme of *B. garinii*. The ADI enzyme plays a critical role in the urea cycle by catalyzing the conversion of arginine to citrulline and ammonia. Given that arginine is necessary for protein synthesis in bacteria, ADI inhibition presents a promising drug target to curtail bacterial growth. The utilization of a docking-based screening approach enabled the rapid screening of marine microbial inhibitors, as it is a streamlined method for identifying potential inhibitors for therapeutic applications.

## 2. Material and Methods

### 2.1. Pan-Proteomics

The proteome sequences of *B. garinii* (*n* = 37) were retrieved in FASTA format from the BV-BRC database (https://www.bv-brc.org/; accessed 30 March 2023) and subjected to pan-proteome analysis using BPGA software [32]. BPGA performs sequence data preprocessing and clustering using USEARCH [33] to generate a pan, unique, and core-genome file. It also performs functional annotation and classification of genes. It uses MUSCLE [34] and rsvg-convert dependencies to align sequences and generate trees based on pan and core genes. Plots were visualized with the aid of gnuplot libraries [35].

### 2.2. Therapeutic Target Mapping

To identify paralogous sequences, the core proteome of *B. garinii* was subjected to the CD-HIT suite [36], with default parameters except for a threshold value of 60%. The CD-HIT suite is a popularly employed software for comparing and clustering protein and genomic sequences to eliminate redundant proteins. To identify essential proteins, BLAST was used against the Database of Essential Genes (DEG) [37] and Database of Essential Gene Clusters (CEG) [38]. Next, the obtained proteins were subjected to a BLAST search against the human proteome (https://www.uniprot.org/uniprotkb?query=reviewed%3Atrue+AND+proteome%3Aup000005640; accessed 4 April 2023) to obtain a dataset of nonhomologous proteins that could be targeted without impacting the human host. For this purpose, the E-value criterion was set at 5∗10^−3^. The resulting dataset was further evaluated against human gut flora, and an E-value greater than 10^−4^ was considered [39]. The final dataset was subjected to a BLAST search against DrugBank [40], with an E-value of <10^−3^. Hits obtained were classified as drug targets. ADI (Accession: WP_031505634.1) was retained for downstream analysis due to its attributes of druggability, uniqueness, and significance in bacterial survival. To assess its similarity to pathogenically important *Borrelia* spp., its sequence was aligned using the multialign server (URL: http://multalin.toulouse.inra.fr/multalin/cgi-bin/multalin.pl; accessed 3 September 2023) with the ADI of the *B. afzelii* and *B. burgdorferi*.

### 2.3. Structural Modeling

The protein 3D model was built with iterative threading assembly refinement (I-TASSER) and AlphaFold [41]. The I-TASSER (https://zhanggroup.org/I-TASSER/; retrieved 8 April 2023) algorithm utilizes the identified templates to build a preliminary model, followed by iterative rounds of refinement that optimize the model structure by minimizing energy and improving stereochemistry [42]. During refinement, the algorithm also generates multiple models, which are ranked based on their quality scores. The top modeled structure was superimposed onto its templates through the use of the FATCAT algorithm within the RCSB pairwise structural alignment server module (https://www.rcsb.org/alignment; accessed 3 August 2023). This was to see the common folds and structural regions of similarity. Another tool, the AlphaFold, deploys deep learning and neural networks to predict the 3D arrangement of a protein, providing valuable insights into its folding and overall structural characteristics. UniRef90 was used for generating multiple sequence alignments. The predicted LDDT (pLDDT) was used for intradomain confidence, whereas Predicted Aligned Error (PAE) was used for determining “between domain” or “between chain” structural confidence. The 3D structure validation was carried out using Ramachandran plot analysis [43, 44], and the best predicted structure was retained for further analysis. The active site was defined as having a residue − specific ligand − binding probability > 0.75, estimated by SVM in I-TASSER. The secondary structure was identified by the ProMotif program [45].

### 2.4. Virtual Screening

The 3D protein structure was prepared for docking as described previously [46, 47]. Seven residues (GLY219, ARG236, HIS272, ASP274, GLY393, ARG394, and CYS399) were defined as active site residues, as predicted by the I-TASSER [42]. S-Nitroso-L-homocysteine was taken as the control, as it is a potent active-site-directed, irreversible inhibitor of ADI (EC number: 3.5.3.6) [48]. It was taken as a control for docking validation, by binding in the pocket and comparing its binding affinity value with new leads. A marine microbial metabolite (lead-like) set of compounds (*n* = 4730) was obtained from the CMNPD website (https://cmnpd.org/; accessed 12 April 2023). It was filtered based on drug-likeliness and lead-like criteria. Drug-like criteria comprised of the “Lipinski Rule of Five” [49], fulfilling the criteria of “molecular weight < 500 Daltons, octanol-water partition coefficient (logP) < 5, hydrogen bond donors < 5, and hydrogen bond acceptors < 10.” A compound that violated more than one of these criteria may have lower oral bioavailability and may face challenges in becoming an effective drug. Hence, the compounds fulfilling at least three of these parameters were selected. In addition, Oprea et al.'s lead-like criteria [50] were checked, and apart from molecular weight and hydrogen bond donors (*n* ≤ 8)/acceptors (*n* ≤ 5), key descriptors included rotatable bonds, rigid bonds, ring count, and logP (-3.5 to 4.5). The rules are aimed at maintaining focus towards effective and orally absorbable compounds. Only molecules fulfilling Lipinski's drug-like and Oprea's lead-like criteria were taken for docking. The docking was performed using AutoDock Vina software, according to the previously described parameters [51]. The affinity value was obtained for best binding pose, the top-ranking compounds were visually inspected, and 2D interactions were mapped to identify the interacting residues. Furthermore, Molecular Mechanics/Poisson-Boltzmann Surface Area (MM/PBSA) values were computed for these complexes, according to Basharat et al. [52]. MM/PBSA is a computational approach used in molecular dynamics simulations to estimate the free energy of binding for protein-ligand complexes. The MM/PBSA method combines molecular mechanics (MM) calculations, which describe the energy associated with the molecular structure, with solvation free energy computations based on the Poisson-Boltzmann equation and the solvent-accessible surface area (PBSA). The relevance of MM/PBSA values lies in their ability to offer insights into the strength of interactions between a ligand and its target protein, helping in the identification and ranking of potential drug candidates.

### 2.5. Pharmacokinetic Profiling

ADMET (Absorption, Distribution, Metabolism, Excretion, and Toxicity) was studied using the pKCSM server (https://biosig.lab.uq.edu.au/pkcsm/theory; accessed 25 April 2023) and SWISS-ADME [53] (http://www.swissadme.ch/index.php; accessed 28 April 2023). Multiple models have been implemented in these tools, including models for predicting human ether-a-go-go-related gene (hERG) inhibition to assess cardiac arrhythmia, cytochrome P450 (CYP) inhibition (leading to possible adverse reactions or therapeutic failures), mutagenicity (Ames test), blood-brain barrier penetration, cytotoxicity, and Caco2 permeability (measure intestinal absorption).

### 2.6. Molecular Dynamics (MD) Simulation

The top-scoring compound along with the control (S-nitroso-L-homocysteine) was subjected to MD simulation to further evaluate stability of the binding interactions with the ADI protein. Docked complexes were prepared using the preparation wizard of Desmond (Schrodinger, LLC, NY, USA) according to previously described parameters [54]. Het states were generated using the Epik module, and heavy atoms were converged to energy minimization till a value of 0.30 Å was attained. The OPLS3e force field and TIP3P water model were selected. The complexes were simulated for 100 ns, and then, the simulation results were analyzed using built-in tools for obtaining RMSD, etc. This allowed insights into the structural and dynamic properties of the complexes. Snapshots at 0, 50, and 100 ns were extracted, and MM/PBSA values were calculated [52].

## 3. Results

### 3.1. Pan-Proteome Analysis

**Figure 1:**
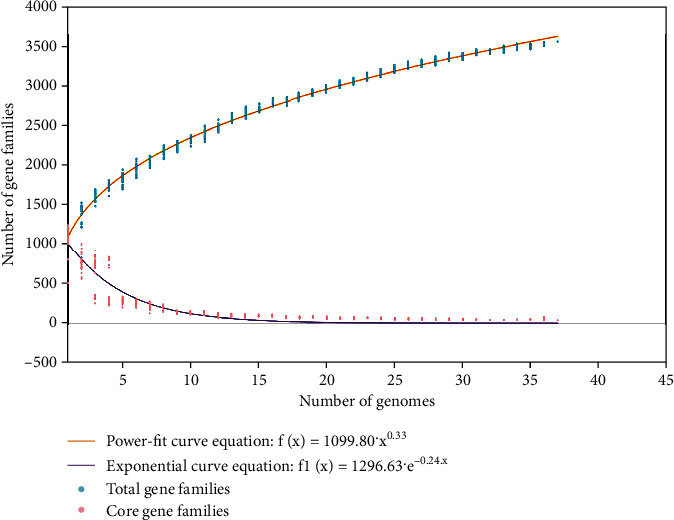
Pan-proteome power curve graph of the studied *B. garinii* strains.

The expected size of the pan-proteome was 3556, while the estimated size was 3622.33. The actual nonredundant proteins in the accessory fraction were 2396. The expected and estimated sizes of the pan-proteome were relatively close, but the actual number of nonredundant proteins in the accessory fraction was lower than expected. This could be due to the reason that some of the proteins in the accessory fraction may be redundant or may not have been detected in the study. The power law model provides a mathematical framework for understanding the open nature of the pan-genome or proteome. It allows the prediction of the pan-proteome size for a given species, even if the genomes of all of the organisms in that species have not yet been sequenced. The equation *f*(*x*) = *a* · *x*^*b*^ is used for this purpose, where *a* and *b* are constants that determine the shape of the curve. The value of the exponent *b* in the power law model determines whether the pan-proteome is open or closed. BPGA determined the value of *b* for *B. garinii* strains as 0.33, indicating that the pan-proteome is open (Figure 1). It signifies that the entirety of proteins within *B. garinii* is not final and that the ongoing advancements in scientific techniques may lead to the continual discovery of new proteins, rendering the catalog of proteins dynamic [55]. Additionally, horizontal gene transfer, etc., could also add to the existing gene pool [56]. The concept of an open proteome becomes particularly pertinent in the contemporary era of high-throughput sequencing and advanced proteomic methodologies, which help identify and elucidate novel proteins, as well as explore various isoforms, thereby contributing to the evolving understanding of the intricate protein landscape within living organisms. Most microorganisms have an open proteome to some extent, as technological advancements and ongoing research efforts continually reveal new protein-coding genes, splice variants, and posttranslational modifications [57]. The number of accessory proteins varied across proteomes, ranging from 444 in IPT107 to 1210 in IPT134 (Table 1). The expected size of the core genome was 0, while the estimated size was 0.18, which suggests significant variability in the genes considered to be part of the core genome. However, the real set of conserved proteins across all strains included in the study was 37, which were expected to be essential for the survival and function of the species.

The number of unique proteins also varied across proteomes, ranging from one in the strain SZ to 69 in IPT128. The total number of unique proteins was 1123. The highest number of unique genes was 69 in IPT128, suggesting that this strain has undergone significant genomic changes compared to other strains. The lowest number, i.e., just one unique protein, was in the strain IPT133, IPT136, IPT140, and SZ (Table 1). Findings indicate that each strain had a unique set of proteins that distinguished it from the other strains. The highest number of exclusively absent genes was 43 for IPT107, suggesting that this strain has lost a significant number of genes compared to other strains. This could be due to factors such as gene deletion or mutation.

### 3.2. Therapeutic Target Mapping

**Figure 2:**
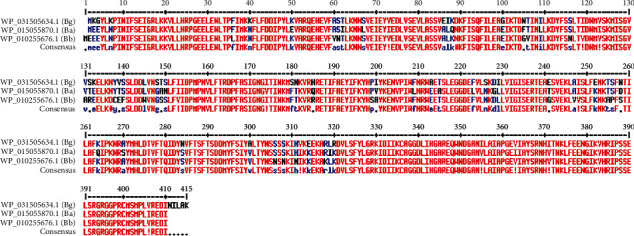
Multiple sequence alignments of the drug target in *B. garinii* and pathogenically important *Borrelia* spp. Bg: *B. garinii*; Ba: *B. afzelii*; Bb: *B. burgdorferi.*

The core proteins were taken and subjected to a subtractive proteomic approach. Among these, no paralogs were present, showing there are no duplicated genes within the core proteome of this bacterium. A total of 25 essential proteins were identified after conducting BLAST searches with CEG and 27 after BLAST against DEG. Altogether, 25 proteins common to both databases were retained (Table 2). Subtraction from the human proteome left only eight sequences, while subtraction from the gut bacterial proteome left only a single protein, i.e., ADI (accession no.: WP_031505634.1) for further analysis. This protein was also identified as a drug target after BLAST with the DrugBank. Its alignment with pathogenically important *B. afzelii* and *B. burgdorferi* species revealed that 17.8% of residues in the consensus sequence had a similarity lower than 50% (Figure 2). Nonetheless, on the whole, the sequence remained conserved.

### 3.3. Structure Modeling of ADI

With regard to the total number of residues in the selected target, the ADI sequence was 414. Out of these, 20 were glycine and 14 were proline residues. These amino acids have been reported distinctly as they play a specific role in protein structure and function, transforming it into fibril or elastomer [58]. Small size and flexibility of glycine make it suitable for regions requiring structural adaptability and attain conformational flexibility. The cyclic structure of proline introduces constraints and rigidity, influencing protein folding and stability. It acts as a “helix breaker” and disrupts regular alpha-helix formations. This disruption is essential to prevent overly rigid structures and to facilitate proper protein folding [59].

**Figure 3:**
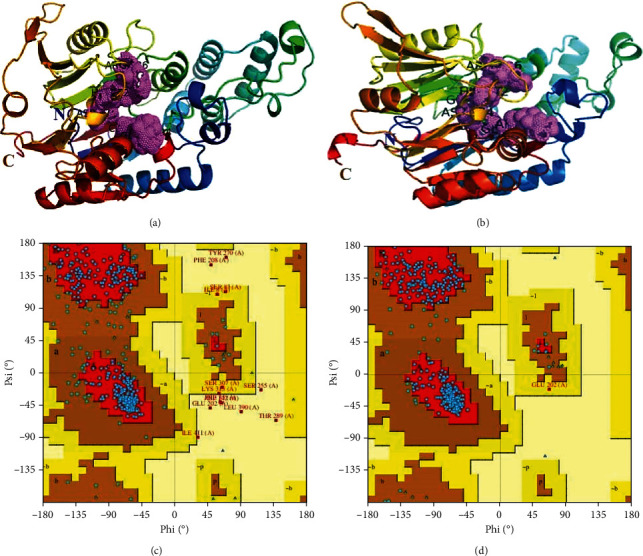
(a) 3D visual representation of the ADI by I-TASSER, showing helices, sheets, and loops. The active site is shown in violet, with dotted residues. The N-terminus is shown with blue N, the -C terminus with brown C. (b) 3D visual representation of the ADI by AlphaFold, showing helices, sheets, and loops. The active site is shown in violet, with dotted residues. The N-terminus is shown with blue N, the -C terminus with brown C. (c) Ramachandran plot of ADI by I-TASSER, depicting backbone dihedral angles of amino acid residues. (d) Ramachandran plot of ADI by AlphaFold, depicting backbone dihedral angles of amino acid residues.

The top threading templates by I-TASSER included ADI from group A *Streptococcus* (PDB ID: 4BOF), enolase from *Mycoplasma pneumoniae* (PDB ID: 7E2Q), ADI from *Mycoplasmopsis arginini* (PDB ID: 1S9R; 1LXY), and the structural protein VP3 from *Bombyx mori* cypovirus 1 (PDB ID: 7WHP). The top five predicted structures had confidence scores ranging from -3 to 0.97. The top-scoring structure (Figure 3(a)) with a confidence score of 0.97 had an estimated TM score of 0.85 ± 0.08 and RMSD of 4.8 ± 3.2 Å. This structure was also overlaid on the top threading templates used for building its coordinates and showed a 48% identity with ADI of group A *Streptococcu*s, 37% identity with ADI of *Mycoplasmopsis arginini*, just 8% with cypovirus 1, and 4% with the enolase of *Mycoplasma pneumoniae*. The predicted secondary structure and solvent accessibility of the sequence suggests that it is a globular protein with a predominantly helical and coil structure. The 3D structure had six sheets, four beta-alpha-beta units, six beta-hairpins, seven beta-bulges, 18 strands, 19 helices, 17 helix-helix interactions, 45 beta-turns, and 16 gamma-turns. The predicted solvent accessibility advocates that the ADI comprises regions partially exposed to the solvent, potentially indicating the presence of binding or interaction sites. The structure predicted by AlphaFold (Figure 3(b)) comprised of 6 sheets, 4 beta-alpha-beta units, 6 beta-hairpins, 6 beta-bulges, 19 strands, 21 helices, 25 helix-helix interactions, 36 beta-turns, and 4 gamma-turns.

The Ramachandran plot statistics by I-TASSER depicted 77.0% residues in the most favored regions, 19.6% in additional allowed regions, 1.1% in the generously allowed regions, and 2.4% in disallowed regions (Figure 3(c)). The model had a reasonable stereochemical quality, with most residues falling in the most favored regions of the Ramachandran plot. However, some dihedral angles in the model were unusual, particularly Omega. The model by AlphaFold had 92.3% residues in the most favored regions, 7.4% in additional allowed regions, 0.3% in generously allowed regions, while none in the disallowed region (Figure 3(d)). pLDDT showed a very high confidence score of >90 and low predicted aligned error for most of the structure. Hence, this structure was used for molecular docking and further analysis.

### 3.4. Docking

**Figure 4:**
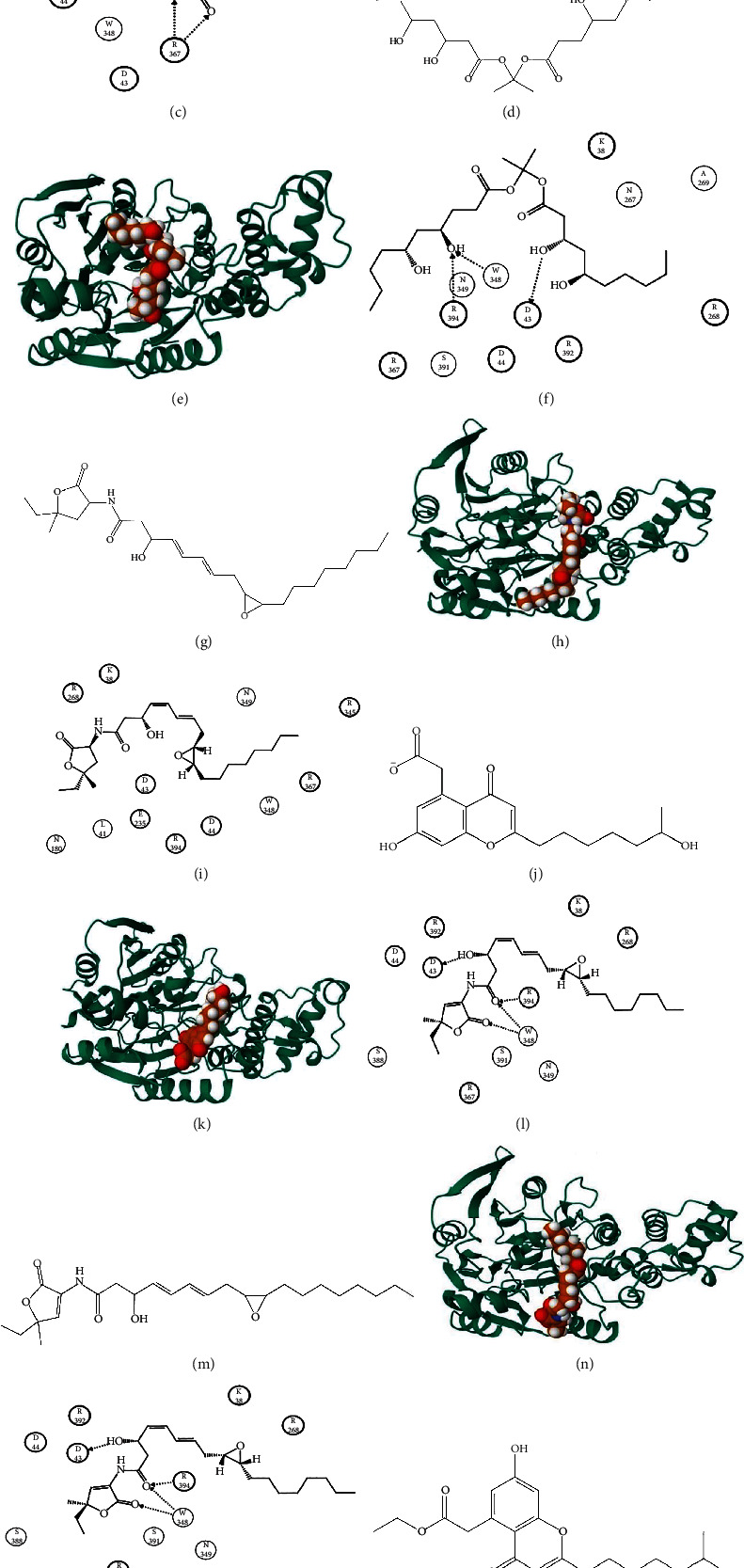
(a) 2D depiction of control (S-nitroso-L-homocysteine). (b) 3D interaction of ADI-control. (c) 2D interaction of ADI-control complex. (d) 2D depiction of CMNPD18759. (e) 3D interaction of ADI-CMNPD18759. (f) 2D interaction of ADI-CMNPD18759 complex. (g) 2D depiction of CMNPD24419. (h) 3D interaction of ADI-CMNPD24419. (i) 2D interaction of ADI-CMNPD24419 complex. (j) 2D depiction of CMNPD24876. (k) 3D interaction of ADI-CMNPD24876. (l) 2D interaction of ADI-CMNPD24876 complex. (m) 2D depiction of CMNPD8737. (n) 3D interaction of ADI-CMNPD8737. (o) 2D interaction of ADI-CMNPD8737 complex. (p) 2D depiction of CMNPD23643. (q) 3D interaction of ADI-CMNPD23643. (r) 2D interaction of ADI-CMNPD23643 complex.

Several inhibitors were prioritized after molecular docking (Table 3) with ADI, having better binding scores than control (S-nitroso-L-homocysteine). Ten residues of ADI interacted with the control (Figures 4(a)–4(c)), 12 with CMNPD18759 (Figures 4(d)–4(f)), 12 with CMNPD24419 (Figures 4(g)–4(i)), nine with CMNPD24876 (Figures 4(j)–4(l)), 11 with CMNPD8737 (Figures 4(m)–4(o)), and ten with CMNPD23643 (Figures 4(p)–4(r)).

The control made four, CMNPD18759 made three, CMNPD24876 made three, CMNPD8737 made four, and CMNPD23643 made five hydrogen bonds. CMNPD24876 also made two arene-hydrogen and one arene-cation bond. Five residues (ASP43, ASP44, ASN349, ARG367, and ARG394) made interactions with all ligands, including the control. MM/PBSA values were computed for protein-ligand complexes, representing the estimated free energy of binding for each compound with ADI. Negative MM/PBSA values generally indicate favorable binding interactions, with an increasing negative value suggesting a stronger binding affinity between the ligand and the protein. The ranking of screened compounds based on the free energy of binding mirrored their order from the docking scores. This consistency is promising and indicates the reliability of both approaches in predicting the binding affinity of the compounds. Control compound was an exception, where a positive value was obtained in contrast to its lower docking score. This discrepancy in docking and MM/PBSA score might be attributed to the different aspects captured by each method and emphasizes the importance of considering multiple computational approaches in drug discovery.

The top-scoring compound aureobasidin (Table 3) is an antibiotic and has previously been implicated as an inhibitor of the inositolphosphorylceramide synthase AUR1 of fungus [60]. It has been isolated from *Aureobasidium pullulans* R106, a black yeast-like fungus [61]. This fungus has previously been isolated from marine sources as well [62]. Korormicin is a metabolite of *Pseudoalteromonas* sp. [63] and has also been known to produce reactive oxygen species to kill bacterial species like *Vibrio cholerae* and *Pseudomonas aeruginosa* [64]. 6′-Hydroxypestalotiopsone C is derived from the mangrove-derived endophytic fungus *Acremonium strictum* [65], while Pestalotiopsone E is derived from *Pestalotiopsis* sp. [66]. Pestalotiopsone has previously shown influenza virus neuraminidase inhibition activity [67]. These compounds depicted good binding efficacy against ADI of *B. garinii* and suggest that these marine-derived compounds could potentially be developed into new therapeutics against Lyme infection.

### 3.5. ADMET

**Figure 5:**
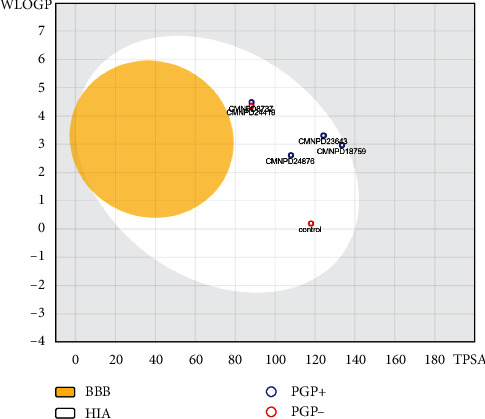
Boiled egg plot showing various ADMET properties of prioritized compounds. BBB: blood-brain barrier; HIA: human intestinal absorption; PGP: P-glycoprotein.

Absorption results by pKCSM showed that the five prioritized marine microbial metabolites had low water solubility but relatively high Caco2 permeability and intestinal absorption (human), suggesting that they may be able to pass through the intestinal lining and enter the bloodstream. This was also confirmed by SWISS-ADME results (Figure 5). The skin permeability values suggest that these compounds may have difficulty penetrating the skin. They were also classified as P-glycoprotein substrates, indicating that they may be transported out of cells by this protein. Additionally, two of these compounds were also predicted as P-glycoprotein inhibitors (Table 4). The steady-state volume of distribution (VDss) values for all five metabolites were negative, indicating that they do not tend to concentrate in tissues, but rather in plasma. The fraction-unbound values were generally low, suggesting that these metabolites tend not to diffuse or traverse cellular membranes. Blood-brain barrier (BBB) permeability values suggest that the metabolites had poor ability to cross the blood-brain barrier, while CNS permeability values suggest that the compounds would have difficulty penetrating the central nervous system. This was again confirmed by SWISS-ADME results (Figure 5). The compounds were not a substrate for cytochrome P (CYP) enzymes, except for CMNPD8737, which had the tendency to be metabolized by CYP3A4. The clearance values for all five metabolites indicate that biliary and hepatic clearance mechanisms may be involved, but there does not seem to be any involvement of the renal OCT2 transporter clearance mechanism in excretion. Regarding toxicity, none of the metabolites were found to be toxic or inhibit potassium channels encoded by hERG (Table 4). Hence, they would not lead to long QT syndrome or subsequent ventricular arrhythmia. However, one of the metabolites, CMNPD8737, was found to be hepatotoxic. The values representing the dose indicate that CMNPD8737 and CMNPD23643 are relatively more toxic for humans, compared to other metabolites having positive values on a log scale. However, for rats, this was not the same, and acute toxicity values were relatively similar for most of the compounds. Only CMNPD18759 had a lower toxicity and a high LD50. The chronic toxicity pattern of the compounds varied from acute toxicity, and CMNPD8737 showed the highest chronic toxicity potential. Studying the toxicity of drug compounds in the ciliated model organism *T. pyriformis* and minnows is essential to assess their environmental impact, ensure regulatory compliance, and better understand their pharmacological and toxicological properties. The control, CMNPD18759, and CMNPD23643 showed higher toxicity in *T. pyriformis*, while CMNPD8737 and CMNPD18759 showed higher toxicity in minnow.

### 3.6. MD Simulation

**Figure 6:**
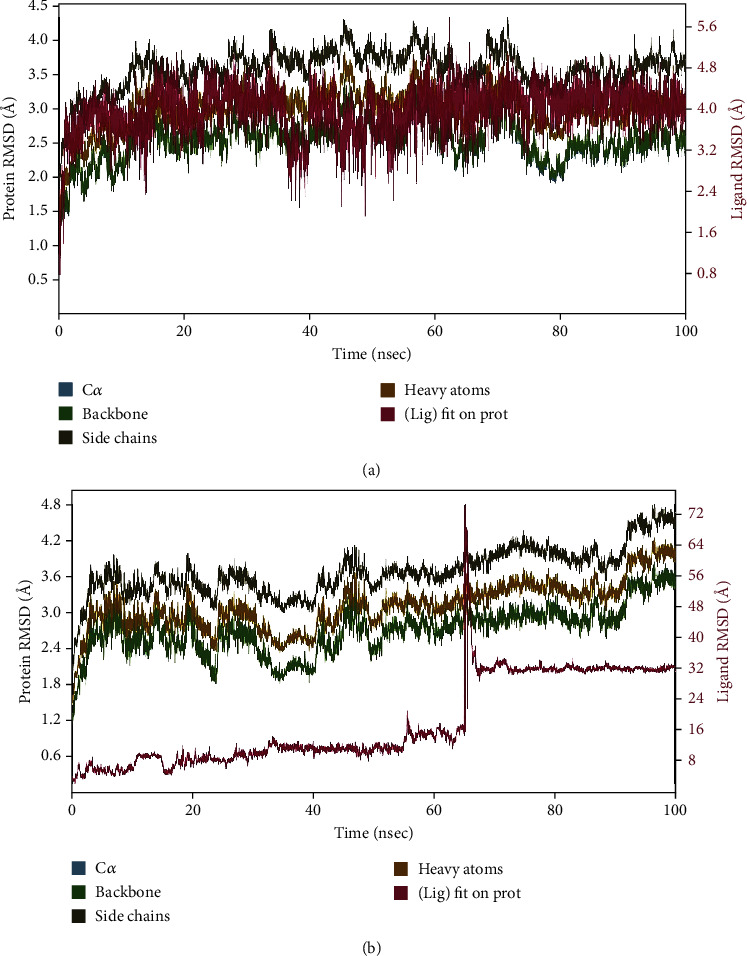
(a) RMSD plot of control compound complexed with ADI. (b) RMSD plot of ADI complexed with CMNPD18759.

The top-scoring compound CMNPD18759 was simulated alongside the control, for 100 ns, to determine its binding stability. The default ensemble used for protein-ligand simulations in Desmond was adopted, i.e., the NPT (constant Number of particles, Pressure, and Temperature) ensemble. Thus, the temperature was maintained constant (300 K) throughout the simulation, allowing the system to exchange energy with a heat bath while keeping the particle number fixed. This is suitable for simulating biological macromolecules like proteins and bound ligands because the conditions encountered in experimental settings are mimicked, where the system is maintained at a constant temperature. Counterions like Na+ and Cl- were introduced to neutralize the net charge of the system. The simulation results suggest significant differences in the binding behavior between the control and CMNPD18759, where the control remained bound stably to the ADI throughout the simulation (Figure 6(a)), while CMNPD18759 underwent a conformational change after 60 ns (Figure 6(b)), which implies for larger scale simulations to attain equilibrium. Side chains showed maximum deviation, followed by heavy atoms and then backbone residues. For investigating conformational changes of CMNPD18759, snapshots of ligand-bound ADI were extracted from the trajectory at the first frame (corresponding to 0 ns), 1000^th^ frame (corresponding to 16 ns), 3000^th^ frame (corresponding to 48 ns), 4000^th^ frame (corresponding to 64 ns), and 5000^th^ frame (corresponding to 80 ns). These were then visualized in comparison to the first frame (reference). The ligand position was altered in due course of time (Supplementary Figure 1). RMSF plot of the control showed flexibility around residue 130 and 260 (reaching up to 5 Å) (Supplementary Figure 2A), while the B-factor remained less than 100 throughout the simulation. The N-terminal and C-terminal regions usually exhibit higher levels of fluctuation compared to other segments of the protein. In contrast, secondary structural elements like alpha-helices and beta-strands tend to display greater rigidity in comparison to the unstructured regions of the protein, resulting in relatively lower fluctuations when considering the loop regions. TRP348, ASN349, ASP350, ARG367, SER391, and GLY393 interacted with the control for more than 30% of the simulation time (Supplementary Figure 2B). ARG367 retained hydrogen bonding for the maximum time, amongst other binding residues (Supplementary Figure 2C). Hydrogen-bonding characteristics are of paramount significance in drug design due to their potent impact on drug specificity, metabolism, and absorption. RMSF of CMNPD18759 binding with ADI was more flexible (Supplementary Figure 3A). There were 24.62% helices and 18.87% strands (overall 43.49% secondary structure elements) in the control trajectory over 100 ns, while CMNPD18759 showed 25.29% helices, 18.31% strands, and 43.60% overall secondary structure elements in the simulation trajectory. No residue retained contact for more than 30% of the simulation time with CMNPD18759. It also formed more hydrophobic interactions than the control (Supplementary Figure 3B). The simulations were replicated, and similar results were observed for both complexes (Supplementary Figure 4).

The MM/PBSA values of the simulated complexes were recorded at different time points (0 ns, 50 ns, and 100 ns). In the case of the control-ADI complex, the values were found to be -30.48 kcal/mol at 0 ns, -32.13 kcal/mol at 50 ns, and -31.85 kcal/mol at 100 ns. For the CMNPD18759-ADI complex, the corresponding MM/PBSA values were -30.44 kcal/mol, -33.38 kcal/mol, and -33.38 kcal/mol at 0 ns, 50 ns, and 100 ns, respectively. These values represent the calculated binding-free energies for each complex at the specified time intervals during the MD simulation. The negative values indicate a favorable interaction, with lower energies as the simulation time proceeds, suggesting stronger binding affinity between the ligand and ADI. The consistency in these values for the CMNPD18759-ADI complex over time suggests a stable and strong protein-ligand affinity.

## 4. Discussion

The field of drug design has been revolutionized by the advent of pan-proteomics, which revolves around the entire coding DNA sequence repertoire of a given microbial species to gain a comprehensive understanding of genetic diversity [68]. This approach enables the identification of core and accessory protein fractions, which can inform the design of drugs that target specific pathways and virulence factors in the select fraction. This approach has previously been implemented for the identification of highly specific and effective therapeutic targets using computational approaches [69, 70]. Using this approach, a single drug target was predicted from the core proteome of 61 strains in the case of *Helicobacter pylori*, a gastric cancer-causing bacterium [71]. Fereshteh et al. used this approach to determine common drug targets in four gram-negative superbugs [72] while Uddin and Jamil used a similar approach to find drug targets in *P. aeruginosa* [73]. Basharat et al. used a similar strategy to mine targets in *Yersinia pseudotuberculosis* [39] and *Shigella* sp. [52, 74]. Here, we used this approach coupled with subtractive proteomics for *B. garinii*, to help prioritize candidate therapeutic targets. This bacterium is responsible for Lyme disease, a tick-borne infectious disease. The primary treatment for Lyme disease is antibiotics, such as doxycycline, amoxicillin, and cefuroxime [75]. However, the emergence of antibiotic-resistant strains is a growing concern, as it limits the effectiveness of existing antibiotics and poses a significant threat to public health [15, 76, 77]. To treat such resistant infection and replenish the drying antibiotic pipeline, we need to identify new therapeutic targets and antibiotics.

Marine-derived metabolites are produced by marine organisms such as algae, microbes, sponges, and corals and have shown promising therapeutic potential in the treatment of various diseases, including infectious diseases [78]. Researchers can more efficiently identify and validate new therapeutic targets and design or optimize novel antibiotics from marine sources, for the treatment of infectious diseases (like Lyme disease). This can be done using molecular docking-aided virtual screening. Herein, we scanned more than 4500 lead-like compounds from marine microbes by leveraging the power of *in silico* methods. This procedure was used to identify lead compounds that can bind and inhibit the function of the selected ADI target. Richards et al. have previously identified its role in *Borrelia* sp. growth, where it boosts intracellular L-arginine crucial for growth [79]. Inhibiting ADI has several advantages as a therapeutic strategy. First, ADI is essential for bacterial growth, so inhibiting ADI can be expected to be effective against a wide range of bacteria. Second, ADI is not present in humans, so inhibiting ADI is unlikely to have any harmful side effects.

**Figure 7:**
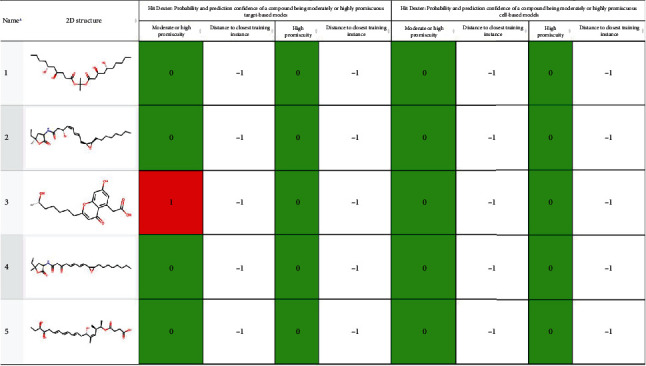
Nonpromiscuous ligands used for inhibition analysis. 1: CMNPD18759; 2: CMNPD24419; 3: CMNPD24876; 4: CMNPD8737; 5: CMNPD23643. Identified from Hit Dexter 3 (URL: https://nerdd.univie.ac.at/hitdexter3/; accessed on 18 September 2023).

*In silico* methods have played a crucial role in identifying and optimizing potential small molecule inhibitors from various sources, including marine-derived products, offering a rapid and cost-effective approach to drug development [80–82]. Marine natural products have garnered significant attention in drug discovery due to their structural diversity and bioactive properties. The number of marine natural products identified to date is >40,000 (https://marinlit.rsc.org/; retrieved 30 April 2023). These compounds come from a diverse range of marine sources, including microorganisms, phytoplankton, various types of algae, sponges, cnidarians, bryozoans, molluscs, tunicates, echinoderms, mangroves, and other intertidal plants and microorganisms. Only 15 marine-derived natural compounds have been approved by FDA, till 2022 [83]. We need to identify and study more of these useful natural product scaffolds to fight off the menace of antibiotic resistance and replenish drying antibiotic pipelines. Five such compounds are shortlisted in this study (Figure 7). The prioritized inhibitors of ADI had relatively high Caco2 permeability and intestinal absorption which suggest that they may be able to pass through the intestinal lining and enter the bloodstream. The skin permeability values indicate that these metabolites may have difficulty penetrating the skin, which limits their potential use in topical applications. The negative VDss values indicate that these metabolites do not tend to cause QT syndrome. They also tend to accumulate in plasma rather than tissue, and this can affect their distribution and elimination, as metabolites that accumulate in tissues can lead to toxic effects. Although drugs are specifically designed to produce therapeutic effects in humans, they may also inadvertently result in unintended side effects in other organisms. This can occur when pharmaceuticals enter water bodies through wastewater or when animals are exposed to pharmaceutical residues in the environment [84]. Hence, values for toxicity were calculated for model organisms like *T. pyriformis* and minnow, to help identify potential ecological risks. Compounds showing adverse effects depict a warning sign for potential environmental issues. Accumulation of even mildly toxic compounds over time in the environment can cause detrimental issues like biomagnification. Hence, this aspect needs to be considered, and such pharmaceutical compounds should be appropriately treated from key outlets like hospital wastewater before being released into the environment [85].

*In silico* methods can help researchers identify and optimize small molecule inhibitors with high binding affinity, selectivity, and pharmacokinetic properties [86]. This approach accelerates the drug discovery process, reduces the cost and time required for traditional drug development, and improves the likelihood of success in clinical trials. However, the limitation of this approach is that molecules may behave differently in the cellular environment, and computational simulations cannot fully capture that. Therefore, compounds identified by this method should be validated through *in vitro* or *in vivo* experiments. Moreover, our study only focused on marine-derived microbial compounds, and future research could investigate other natural sources, such as terrestrial plants or fungi, for identifying potential therapeutic compounds. Furthermore, this study only examined a relatively small subset of marine microbes, and a more extensive exploration of marine microbial diversity could yield additional promising compounds.

## 5. Conclusion

There is a critical need for the development of novel antibiotics that are effective against the causative agents of Lyme disease, for instance, *B. garinii*, *B. burgdorferi*, and *B. afzelii*. Achieving this necessitates the identification of new therapeutic targets and the development of inhibitors that can selectively and effectively inhibit these specific targets. In this study, we focused on one bacterium, i.e., *B. garinii*, and used a virtual screening approach to identify marine compounds having inhibitory potential against *B. garinii*. We screened a library of over 4000 marine compounds against the ADI enzyme and identified several compounds with good binding and possible inhibition activity. We conclude that marine compounds are a rich source of novel leads for drug development against *B. garinii*. These compounds may be further evaluated *in vitro* and *in vivo*, but our findings provide a promising starting point for the development of new antibiotics that are urgently needed to combat Lyme disease.

## Figures and Tables

**Table 1:** Pan-proteome statistics for 37 *B. garinii* strains studied.

Genome no.	Organism name	No. of core genes	No. of accessory genes	No. of unique genes	No. of exclusively absent genes
1	IPT105	37	1169	14	0
2	IPT107	37	444	30	43
3	IPT108	37	1138	22	0
4	IPT113	37	1046	43	0
5	IPT114	37	1051	26	1
6	IPT115	37	1043	41	2
7	IPT117	37	774	34	14
8	IPT120	37	1148	14	0
9	IPT124	37	1181	22	0
10	IPT126	37	926	44	6
11	IPT128	37	1086	69	1
12	IPT129	37	984	46	2
13	IPT130	37	1067	25	0
14	IPT131	37	1139	36	0
15	BgVir	37	765	4	0
16	IPT74	37	1156	55	0
17	IPT75	37	1145	27	0
18	IPT76	37	1168	24	0
19	IPT86	37	1001	52	1
20	IPT88	37	1179	32	1
21	IPT89	37	1171	36	0
22	IPT90	37	1014	48	1
23	IPT91	37	1079	32	3
24	IPT94	37	1142	34	0
25	IPT96	37	1186	7	0
26	IPT98	37	947	49	3
27	IPT99	37	1131	17	3
28	IPT101	37	872	42	15
29	IPT104	37	1160	21	0
30	IPT133	37	1112	20	0
31	IPT134	37	1210	17	0
32	IPT136	37	1053	44	1
33	IPT139	37	1089	26	0
34	IPT140	37	1084	42	1
35	PBi	37	857	19	0
36	PBr	37	1114	8	0
37	SZ	37	769	1	0

**Table 2:** Overview of the essential proteins, including their length and associated KEGG pathway ID.

Serial no.	Length (amino acids)	KEGG pathway ID	Definition
1.	1155	K03043	rpoB; DNA-directed RNA polymerase subunit beta [EC:2.7.7.6]
2.	1124	K03723	mfd; transcription-repair coupling factor (superfamily II helicase) [EC:5.6.2.4]
3.	899	K03070	secA; preprotein translocase subunit SecA [EC:7.4.2.8]
4.	849	K03168	topA; DNA topoisomerase I [EC:5.6.2.1]
5.	810	K02469	gyrA; DNA gyrase subunit A [EC:5.6.2.2]
6.	806	K01338	lon; ATP-dependent Lon protease [EC:3.4.21.53]
7.	791	K03217	Membrane protein insertase YidC
8.	669	K02355	fusA, GFM, EFG; elongation factor G
9.	631	K03086	rpoD; RNA polymerase primary sigma factor
10.	594	K01872	AARS, alaS; alanyl-tRNA synthetase [EC:6.1.1.7]
11.	593	K02316	dnaG; DNA primase [EC:2.7.7.101]
12.	536	K23537	nupA; general nucleoside transport system ATP-binding protein
13.	488	K01881	PARS, proS; prolyl-tRNA synthetase [EC:6.1.1.15]
14.	455	K03106	SRP54, ffh; signal recognition particle subunit SRP54 [EC:3.6.5.4]
15.	452	K02481	rrp-2; response regulatory protein
16.	414	K01478	arcA; arginine deiminase [EC:3.5.3.6]
17.	389	K03438	mraW, rsmH; 16S rRNA (cytosine1402-N4)-methyltransferase [EC:2.1.1.199]
18.	362	K01000	mraY; phospho-N-acetylmuramoyl-pentapeptide-transferase [EC:2.7.8.13]
19.	352	K03588	ftsW, spoVE; cell division protein FtsW
20.	350	K02836	prfB; peptide chain release factor 2
21.	349	K15582	oppC; oligopeptide transport system permease protein
22.	327	K00611	OTC, argF, argI; ornithine carbamoyltransferase [EC:2.1.3.3]
23.	279	K02357	tsf, TSFM; elongation factor Ts
24.	215	K02890	RP-L22, MRPL22, rplV; large subunit ribosomal protein L22
25.	197	K01358	clpP, CLPP; ATP-dependent Clp protease, protease subunit [EC:3.4.21.92]

**Table 3:** Docking and MM/PBSA scores of prioritized metabolites.

Serial no.	Compound	Common name	IUPAC name	Molecular weight (Daltons)	logP (o/w)	Lipinski donor	Lipinski acceptor	Docking score	MM/PBSA values of compound (kcal/mol)	MM/PBSA values of compound complexed with ADI (kcal/mol)
1	Control	S-Nitroso-L-homocysteine	(2*S*)-2-Amino-4-nitrososulfanylbutanoic acid	164.19	-2.0	3	5	-4.6	0.56	-30.23
2	CMNPD18759	Aureobasidin	2-[(4*R*,6*R*)-4,6-Dihydroxydecanoyl]oxypropan-2-yl (3*S*,5*R*)-3,5-dihydroxydecanoate	448.60	4.64	4	8	-5.3	-0.32	-30.27
3	CMNPD24419	Korormicin G	(3*R*,4*Z*,6*E*)-N-[(3*S*,5*S*)-5-Ethyl-5-methyl-2-oxooxolan-3-yl]-3-hydroxy-8-[(2*S*,3*R*)-3-octyloxiran-2-yl]octa-4,6-dienamide	435.61	5.18	2	6	-5.2	-0.38	-30.23
4	CMNPD24876	6′-Hydroxypestalotiopsone C	2-[7-Hydroxy-2-[(6*S*)-6-hydroxyheptyl]-4-oxochromen-5-yl]acetic acid	334.37	2.75	4	6	-5.1	0.10	-30.28
5	CMNPD8737	Korormicin†	(4*Z*,6*E*)-*N*-(5-Ethyl-5-methyl-2-oxofuran-3-yl)-3-hydroxy-8-(3-octyloxiran-2-yl)octa-4,6-dienamide	433.59	5.38	2	6	-4.9	-0.37	-30.41
6	CMNPD23643	Pestalotiopsone E	Ethyl 2-[7-hydroxy-2-[(6*S*)-6-hydroxyheptyl]-4-oxochromen-5-yl]acetate	362.42	3.35	2	6	-4.70	0.01	-30.28

MM/PBSA value of ADI was -30.32 kcal/mol. Descriptor values for Lipinski's rule for druggability of molecules are also provided. All molecules fulfilled Oprea's lead-like criteria as well.

**Table 4:** ADMET properties of prioritized marine bacterial metabolites.

Property	Model name	Control	CMNPD18759	CMNPD24419	CMNPD24876	CMNPD8737	CMNPD23643	Unit
Absorption	Water solubility	-0.5	-3.032	-3.644	-3.644	-3.8	-3.156	Numeric (log mol/L)
	Caco2 permeability	-0.316	0.666	0.537	0.537	0.723	0.429	Numeric (log Papp in 10^−6^ cm/s)
	Intestinal absorption (human)	51.503	52.118	55.147	55.147	92.291	35.358	Numeric (% absorbed)
	Skin permeability	-2.735	-2.729	-2.699	-2.699	-2.748	-2.735	Numeric (log Kp)
	P-Glycoprotein substrate	No	Yes	Yes	Yes	Yes	Yes	Categorical (yes/no)
	P-Glycoprotein I inhibitor	No	Yes	No	No	Yes	No	Categorical (yes/no)
	P-Glycoprotein II inhibitor	No	No	No	No	Yes	Yes	Categorical (yes/no)

Distribution	VDss (human)	-0.656	0.129	-0.064	-0.064	-0.496	-1.218	Numeric (log L/kg)
	Fraction unbound (human)	0.782	0.345	0.146	0.146	0.093	0.285	Numeric (Fu)
	BBB permeability	-0.661	-1.721	-1.099	-1.099	-0.419	-1.426	Numeric (log BB)
	CNS permeability	-3.519	-3.74	-2.881	-2.881	-3.032	-3.387	Numeric (log PS)

Metabolism	CYP2D6 substrate	Yes	No	No	No	No	No	Categorical (yes/no)
	CYP3A4 substrate	No	No	No	No	Yes	No	Categorical (yes/no)
	CYP1A2 inhibitor	No	No	No	No	No	No	Categorical (yes/no)
	CYP2C19 inhibitor	No	No	No	No	No	No	Categorical (yes/no)
	CYP2C9 inhibitor	No	No	No	No	No	No	Categorical (yes/no)
	CYP2D6 inhibitor	No	No	No	No	No	No	Categorical (yes/no)
	CYP3A4 inhibitor	No	No	No	No	No	No	Categorical (yes/no)

Excretion	Total clearance	0.097	1.813	0.944	0.944	1.451	2.16	Numeric (log mL/min/kg)
	Renal OCT2 substrate	No	No	No	No	No	No	Categorical (yes/no)

Toxicity	AMES toxicity	No	No	No	No	No	No	Categorical (yes/no)
	Max. tolerated dose (human)	1.362	0.332	0.367	0.367	-1.485	-1.343	Numeric (log mg/kg/day)
	hERG I inhibitor	No	No	No	No	No	No	Categorical (yes/no)
	hERG II inhibitor	No	No	No	No	No	No	Categorical (yes/no)
	Oral rat acute toxicity (LD50)	1.726	4.178	2.124	2.124	2.377	2.738	Numeric (mol/kg)
	Oral rat chronic toxicity (LOAEL)	1.929	1.983	1.204	1.204	0.747	1.13	Numeric (log mg/kg_bw/day)
	Hepatotoxicity	No	No	No	No	Yes	No	Categorical (yes/no)
	Skin sensitisation	No	No	No	No	No	No	Categorical (yes/no)
	*T. pyriformis* toxicity	0.284	0.285	0.537	0.537	0.542	0.288	Numeric (log *μ*g/L)
	Minnow toxicity	2.278	0.357	0.873	0.873	0.237	1.044	Numeric (log mM)

## Data Availability

All the data used or generated in this study is provided as an accession number or relevant information as tables in the manuscript.

## Supplementary Materials

Supplementary Materials Supplementary Figure 1: protein ligand complex position at various time stages. Water molecules and ions have not been shown to visualize clear placement of the ligand. (A) First frame at 0 ns, showing the ligand in CPK representation. The protein is shown in the ribbon (colored by the secondary structure). (B) 1000th frame at 16 ns, showing the ligand in CPK representation. The protein is shown in the ribbon (purple colored). (C) 3000th frame at 48 ns, showing the ligand in CPK representation. The protein is shown in the ribbon (gray colored). (D) 4000th frame at 64 ns, showing the ligand in CPK representation. The protein is shown in the ribbon (wheat colored). (E) 5000th frame at 80 ns, showing the ligand in CPK representation. The protein is shown in the ribbon (orange colored). Supplementary Figure 2: (A) RMSF of control compound. (B) Residue interaction of control compound, retained for more than 30% of simulation time. (C) Interaction fraction of residues during simulation. Hydrogen bonds are shown in green, hydrophobic in mauve, ionic in pink, and water bridges in blue. Supplementary Figure 3: (A) RMSF of CMNPD18759. (B) Interaction fraction of residues during simulation. Hydrogen bonds are shown in green, hydrophobic in mauve, ionic in pink, and water bridges in blue. Supplementary Figure 4: (A) RMSD plot of control and protein. R1 denotes the first replicate, and R2 denotes the second replicate. (B) RMSD plot of CMNPD18759 and the protein. R1 denotes the first replicate, and R2 denotes the second replicate. (C) RMSF of the control-protein complex. (D) RMSF of the CMNPD18759-protein complex.

## References

[1] Pisarek M., Kalinowski M., Skrzypczak M., Winiarczyk M., Abramowicz B., Winiarczyk S. (2022). Lyme disease in Bernese Mountain dogs. Is it a real problem?. *Polish Journal of Veterinary Sciences*.

[2] Ashour R., Hamza D., Kadry M., Sabry M. A. (2023). The surveillance of *Borrelia* species in *Camelus dromedarius* and associated ticks: the first detection of *Borrelia miyamotoi* in Egypt. *Veterinary Sciences*.

[3] Radolf J. D., Strle K., Lemieux J. E., Strle F. (2021). Lyme disease in humans. *Current Issues in Molecular Biology*.

[4] Boulanger N., Boyer P., Talagrand-Reboul E., Hansmann Y. (2019). Ticks and tick-borne diseases. *Medecine et Maladies Infectieuses*.

[5] Tracy K. E., Baumgarth N. (2017). *Borrelia burgdorferi* manipulates innate and adaptive immunity to establish persistence in rodent reservoir hosts. *Frontiers in Immunology*.

[6] Steere A. C., Strle F., Wormser G. P. (2016). Lyme borreliosis. *Nature Reviews Disease Primers*.

[7] Ganta R. R. (2022). Spirilla I: Borrelia. *Veterinary Microbiology*.

[8] Sanjuna C., Pravalika P., Priya C. S., Venkataswamy M., Ramesh A. (2018). The insidious disease from insects: Lyme disease. *Research Journal of Pharmaceutical Dosage Forms and Technology*.

[9] Schoen R. T. (2020). Challenges in the diagnosis and treatment of Lyme disease. *Current Rheumatology Reports*.

[10] Bush L. M., Vazquez-Pertejo M. T. (2018). Tick borne illness—Lyme disease. *Disease-a-Month*.

[11] Pritt B. S., Mead P. S., Johnson D. K. H. (2016). Identification of a novel pathogenic *Borrelia* species causing Lyme borreliosis with unusually high spirochaetaemia: a descriptive study. *The Lancet Infectious Diseases*.

[12] Cardenas-de la Garza J. A., De la Cruz-Valadez E., Ocampo-Candiani J., Welsh O. (2019). Clinical spectrum of Lyme disease. *European Journal of Clinical Microbiology & Infectious Diseases*.

[13] Chaaya G., Jaller-Char J. J., Ali S. K. (2016). Beyond the bull's eye: recognizing Lyme disease. *Journal of Family Practice*.

[14] Sharma B., Brown A. V., Matluck N. E., Hu L. T., Lewis K. (2015). *Borrelia burgdorferi*, the causative agent of Lyme disease, forms drug-tolerant persister cells. *Antimicrobial Agents and Chemotherapy*.

[15] Di Domenico E. G., Cavallo I., Bordignon V. (2018). The emerging role of microbial biofilm in lyme neuroborreliosis. *Frontiers in Neurology*.

[16] Sapi E., Balasubramanian K., Poruri A. (2016). Evidence of *in vivo* existence of *Borrelia* biofilm in borrelial lymphocytomas. *European Journal of Microbiology and Immunology*.

[17] Anderson C., Brissette C. A. (2021). The brilliance of *Borrelia*: mechanisms of host immune evasion by Lyme disease-causing spirochetes. *Pathogens*.

[18] Sharma D., Sharma A., Singh B., Verma S. K. (2021). Pan-proteome profiling of emerging and re-emerging zoonotic pathogen *Orientia tsutsugamushi* for getting insight into microbial pathogenesis. *Microbial Pathogenesis*.

[19] Lavecchia A., Cerchia C. (2016). In silico methods to address polypharmacology: current status, applications and future perspectives. *Drug Discovery Today*.

[20] Kumar A., Tiwari A., Sharma A. (2018). Changing paradigm from one target one ligand towards multi-target directed ligand design for key drug targets of Alzheimer disease: an important role of in silico methods in multi-target directed ligands design. *Current Neuropharmacology*.

[21] Basharat Z., Khan K., Jalal K., Alnasser S. M., Majeed S., Zehra M. (2022). Inferring therapeutic targets in *Candida albicans* and possible inhibition through natural products: a binding and physiological based pharmacokinetics snapshot. *Life*.

[22] Wiese J., Imhoff J. F. (2019). Marine bacteria and fungi as promising source for new antibiotics. *Drug Development Research*.

[23] Ruocco N., Costantini S., Guariniello S., Costantini M. (2016). Polysaccharides from the marine environment with pharmacological, cosmeceutical and nutraceutical potential. *Molecules*.

[24] Nweze J. A., Mbaoji F. N., Huang G. (2020). Antibiotics development and the potentials of marine-derived compounds to stem the tide of multidrug-resistant pathogenic bacteria, fungi, and protozoa. *Marine Drugs*.

[25] Jensen P. R., Moore B. S., Fenical W. J. (2015). The marine actinomycete genus *Salinispora*: a model organism for secondary metabolite discovery. *Natural Product Reports*.

[26] Ogawara H. (2019). Comparison of antibiotic resistance mechanisms in antibiotic-producing and pathogenic bacteria. *Molecules*.

[27] Lee Y., Phat C., Hong S.-C. (2017). Structural diversity of marine cyclic peptides and their molecular mechanisms for anticancer, antibacterial, antifungal, and other clinical applications. *Peptides*.

[28] Kurtböke D. İ., Grkovic T., Quinn R. J., Kim S. K. (2015). Marine Actinomycetes in Biodiscovery. *Springer Handbook of Marine Biotechnology. Springer Handbooks*.

[29] Li R. (2016). Marinopyrroles: unique drug discoveries based on marine natural products. *Medicinal Research Reviews*.

[30] Chen X., Chen J., Yan Y. (2019). Quorum sensing inhibitors from marine bacteria *Oceanobacillus* sp. XC22919. *Natural Product Research*.

[31] Borges A., Simões M. (2019). Quorum sensing inhibition by marine bacteria. *Marine Drugs*.

[32] Chaudhari N. M., Gupta V. K., Dutta C. (2016). BPGA-an ultra-fast pan-genome analysis pipeline. *Scientific Reports*.

[33] Edgar R. C. (2010). Search and clustering orders of magnitude faster than BLAST. *Bioinformatics*.

[34] Edgar R. C. (2004). MUSCLE: multiple sequence alignment with high accuracy and high throughput. *Nucleic Acids Research*.

[35] Williams T., Kelley C., Bersch C. (2017). gnuplot 5.2. An interactive plotting program. http://www.gnuplot.info/docs_5.

[36] Huang Y., Niu B., Gao Y., Fu L., Li W. (2010). CD-HIT suite: a web server for clustering and comparing biological sequences. *Bioinformatics*.

[37] Zhang R., Ou H. Y., Zhang C. T. (2004). DEG: a database of essential genes. *Nucleic Acids Research*.

[38] Ye Y.-N., Hua Z.-G., Huang J., Rao N., Guo F.-B. (2013). CEG: a database of essential gene clusters. *BMC Genomics*.

[39] Basharat Z., Jahanzaib M., Yasmin A., Khan I. A. (2021). Pan-genomics, drug candidate mining and ADMET profiling of natural product inhibitors screened against *Yersinia pseudotuberculosis*. *Genomics*.

[40] Wishart D. S., Feunang Y. D., Guo A. C. (2018). DrugBank 5.0: a major update to the DrugBank database for 2018. *Nucleic Acids Research*.

[41] Varadi M., Anyango S., Deshpande M. (2022). AlphaFold protein structure database: massively expanding the structural coverage of protein-sequence space with high-accuracy models. *Nucleic Acids Research*.

[42] Yang J., Yan R., Roy A., Xu D., Poisson J., Zhang Y. (2015). The I-TASSER suite: protein structure and function prediction. *Nature Methods*.

[43] Hooft R. W., Sander C., Vriend G. (1997). Objectively judging the quality of a protein structure from a Ramachandran plot. *Bioinformatics*.

[44] Gopalakrishnan K., Sowmiya G., Sheik S., Sekar K. J. (2007). Ramachandran plot on the web (2.0). *Protein and Peptide Letters*.

[45] Hutchinson E. G., Thornton J. M. (1996). PROMOTIF—a program to identify and analyze structural motifs in proteins. *Protein Science*.

[46] Mehmood A., Khan M. T., Kaushik A. C., Khan A. S., Irfan M., Wei D.-Q. (2019). Structural dynamics behind clinical mutants of PncA-Asp12Ala, Pro54Leu, and His57Pro of *Mycobacterium tuberculosis* associated with pyrazinamide resistance. *Frontiers in Bioengineering and Biotechnology*.

[47] Wadood A., Mehmood A., Khan H. (2017). Epitopes based drug design for dengue virus envelope protein: a computational approach. *Computational Biology and Chemistry*.

[48] Li Z., Kulakova L., Li L. (2009). Mechanisms of catalysis and inhibition operative in the arginine deiminase from the human pathogen *Giardia lamblia*. *Bioorganic Chemistry*.

[49] Chen X., Li H., Tian L., Li Q., Luo J., Zhang Y. (2020). Analysis of the physicochemical properties of acaricides based on Lipinski’s rule of five. *Journal of Computational Biology*.

[50] Oprea T. I., Allu T. K., Fara D. C., Rad R. F., Ostopovici L., Bologa C. G. (2007). Lead-like, drug-like or "pub-like": how different are they?. *Journal of Computer-Aided Molecular Design*.

[51] Huey R., Morris G. M., Forli S. (2012). Using AutoDock 4 and AutoDock vina with AutoDockTools: a tutorial. *The Scripps Research Institute Molecular Graphics Laboratory*.

[52] Basharat Z., Khan K., Jalal K. (2022). An in silico hierarchal approach for drug candidate mining and validation of natural product inhibitors against pyrimidine biosynthesis enzyme in the antibiotic-resistant *Shigella flexneri*. *Infection, Genetics and Evolution*.

[53] Daina A., Michielin O., Zoete V. (2017). SwissADME: a free web tool to evaluate pharmacokinetics, drug-likeness and medicinal chemistry friendliness of small molecules. *Scientific Reports*.

[54] Aziz M., Ejaz S. A., Rehman H. M. (2023). Identification of NEK7 inhibitors: structure based virtual screening, molecular docking, density functional theory calculations and molecular dynamics simulations. *Journal of Biomolecular Structure and Dynamics*.

[55] Carlos Guimaraes L. (2015). Inside the pan-genome - methods and software overview. *Current Genomics*.

[56] Zhao Y., Sun C., Zhao D. (2018). PGAP-X: extension on pan-genome analysis pipeline. *BMC Genomics*.

[57] Brockhurst M. A., Harrison E., Hall J. P., Richards T., McNally A., MacLean C. (2019). The ecology and evolution of pangenomes. *Current Biology*.

[58] Rauscher S., Baud S., Miao M., Keeley F. W., Pomes R. (2006). Proline and glycine control protein self-organization into elastomeric or amyloid fibrils. *Structure*.

[59] Öten A. M., Atak E., Taktak Karaca B., Fırtına S., Kutlu A. (2023). Discussing the roles of proline and glycine from the perspective of cold adaptation in lipases and cellulases. *Biocatalysis and Biotransformation*.

[60] Zhong W., Jeffries M. W., Georgopapadakou N. H. (2000). Inhibition of inositol phosphorylceramide synthase by Aureobasidin A in *Candida* and *Aspergillus* species. *Antimicrobial Agents and Chemotherapy*.

[61] Černoša A., Gostinčar C., Lavrin T., Kostanjšek R., Lenassi M., Gunde-Cimerman N. (2022). Isolation and characterization of extracellular vesicles from biotechnologically important fungus *Aureobasidium pullulans*. *Fungal Biology and Biotechnology*.

[62] Chi Z., Ma C., Wang P., Li H. (2007). Optimization of medium and cultivation conditions for alkaline protease production by the marine yeast *Aureobasidium pullulans*. *Bioresource Technology*.

[63] Dembitsky V. M. (2022). Microbiological aspects of unique, rare, and unusual fatty acids derived from natural amides and their pharmacological profile. *Microbiology Research*.

[64] Zhang Y., Lin M., Qin Y. (2023). Anti-vibrio potential of natural products from marine microorganisms. *European Journal of Medicinal Chemistry*.

[65] Chen S., Cai R., Liu Z., Cui H., She Z. (2022). Secondary metabolites from mangrove-associated fungi: source, chemistry and bioactivities. *Natural Product Reports*.

[66] Blunt J. W., Copp B. R., Keyzers R. A., Munro M. H., Prinsep M. R. (2015). Marine natural products. *Natural Product Reports*.

[67] Luo X., Yang J., Chen F. (2018). Structurally diverse polyketides from the mangrove-derived fungus *Diaporthe* sp. SCSIO 41011 with their anti-influenza A virus activities. *Frontiers in Chemistry*.

[68] Barh D., Soares S. C., Tiwari S., Azevedo V. A. D. C. (2020). *Pan-Genomics: Applications, Challenges, and Future Prospects*.

[69] Broadbent J. A., Broszczak D. A., Tennakoon I. U., Huygens F. (2016). Pan-proteomics, a concept for unifying quantitative proteome measurements when comparing closely-related bacterial strains. *Expert Review of Proteomics*.

[70] Uddin R., Sufian M. (2016). Core proteomic analysis of unique metabolic pathways of *Salmonella enterica* for the identification of potential drug targets. *PLoS One*.

[71] Uddin R., Khalil W. (2020). A comparative proteomic approach using metabolic pathways for the identification of potential drug targets against *Helicobacter pylori*. *Genes & Genomics*.

[72] Fereshteh S., Noori Goodarzi N., Kalhor H., Rahimi H., Barzi S. M., Badmasti F. (2023). Identification of putative drug targets in highly resistant gram-negative bacteria and drug discovery against Glycyl-tRNA synthetase as a new target. *Bioinformatics and Biology Insights*.

[73] Uddin R., Jamil F. (2018). Prioritization of potential drug targets against *P. aeruginosa* by core proteomic analysis using computational subtractive genomics and protein-protein interaction network. *Computational Biology and Chemistry*.

[74] Basharat Z., Jahanzaib M., Rahman N. (2021). Therapeutic target identification via differential genome analysis of antibiotic resistant *Shigella sonnei* and inhibitor evaluation against a selected drug target. *Infection, Genetics and Evolution*.

[75] Eppes S. C., Childs J. A. (2002). Comparative study of cefuroxime axetil versus amoxicillin in children with early Lyme disease. *Pediatrics*.

[76] Limbach F., Jaulhac B., Puechal X. (2001). Treatment resistant Lyme arthritis caused by *Borrelia garinii*. *Annals of the Rheumatic Diseases*.

[77] Steere A. C., Gross D., Meyer A. L., Huber B. T. (2001). Autoimmune mechanisms in antibiotic treatment-resistant Lyme arthritis. *Journal of Autoimmunity*.

[78] Karthikeyan A., Joseph A., Nair B. G. (2022). Promising bioactive compounds from the marine environment and their potential effects on various diseases. *Journal of Genetic Engineering and Biotechnology*.

[79] Richards C. L., Raffel S. J., Bontemps-Gallo S., Dulebohn D. P., Herbert T. C., Gherardini F. C. (2022). The arginine deaminase system plays distinct roles in *Borrelia burgdorferi* and *Borrelia hermsii*. *PLoS Pathogens*.

[80] Mia M. M., Hasan M., Miah M. M., Hossain M. A. S., Islam M. S., Shanta R. N. (2022). Inhibitory potentiality of secondary metabolites extracted from marine fungus target on avian influenza virus-a subtype H5N8 (Neuraminidase) and H5N1 (nucleoprotein): a rational virtual screening. *Veterinary and Animal Science*.

[81] Lever J., Kreuder F., Henry J. (2022). Targeted isolation of antibiotic brominated alkaloids from the marine sponge *Pseudoceratina durissima* using virtual screening and molecular networking. *Marine Drugs*.

[82] Kang N., Heo S.-Y., Cha S.-H., Ahn G., Heo S.-J. (2022). In silico virtual screening of marine aldehyde derivatives from seaweeds against SARS-CoV-2. *Marine Drugs*.

[83] Banerjee P., Mandhare A., Bagalkote V. (2022). Marine natural products as source of new drugs: an updated patent review (July 2018-July 2021). *Expert Opinion on Therapeutic Patents*.

[84] Klatte S., Schaefer H.-C., Hempel M. (2017). Pharmaceuticals in the environment–a short review on options to minimize the exposure of humans, animals and ecosystems. *Sustainable Chemistry and Pharmacy*.

[85] Patel N., Khan M., Shahane S. (2020). Emerging pollutants in aquatic environment: source, effect, and challenges in biomonitoring and bioremediation-a review. *Pollution*.

[86] Ekins S., Mestres J., Testa B. (2007). In silico pharmacology for drug discovery: applications to targets and beyond. *British Journal of Pharmacology*.

