# Supplementary material for: Screening Marine Microbial Metabolites as Promising Inhibitors of Borrelia garinii: A Structural Docking Approach towards Developing Novel Lyme Disease Treatment
Source: Biomed Res Int. 2024 Feb 29;2024:9997082. doi: 10.1155/2024/9997082 (PMC10919988; doi:10.1155/2024/9997082)
Supplement: Supplementary Materials — Supplementary Figure 1: protein ligand complex position at various time stages. Water molecules and ions have not been shown to visualize clear placement of the ligand. (A) First frame at 0 ns, showing the ligand in CPK representation. The protein is shown in the ribbon (colored by the secondary structure). (B) 1000th frame at 16 ns, showing the ligand in CPK representation. The protein is shown in the ribbon (purple colored). (C) 3000th frame at 48 ns, showing the ligand in CPK representation. The protein is shown in the ribbon (gray colored). (D) 4000th frame at 64 ns, showing the ligand in CPK representation. The protein is shown in the ribbon (wheat colored). (E) 5000th frame at 80 ns, showing the ligand in CPK representation. The protein is shown in the ribbon (orange colored). Supplementary Figure 2: (A) RMSF of control compound. (B) Residue interaction of control compound, retained for more than 30% of simulation time. (C) Interaction fraction of residues during simulation. Hydrogen bonds are shown in green, hydrophobic in mauve, ionic in pink, and water bridges in blue. Supplementary Figure 3: (A) RMSF of CMNPD18759. (B) Interaction fraction of residues during simulation. Hydrogen bonds are shown in green, hydrophobic in mauve, ionic in pink, and water bridges in blue. Supplementary Figure 4: (A) RMSD plot of control and protein. R1 denotes the first replicate, and R2 denotes the second replicate. (B) RMSD plot of CMNPD18759 and the protein. R1 denotes the first replicate, and R2 denotes the second replicate. (C) RMSF of the control-protein complex. (D) RMSF of the CMNPD18759-protein complex. [file 9997082.f1.docx]

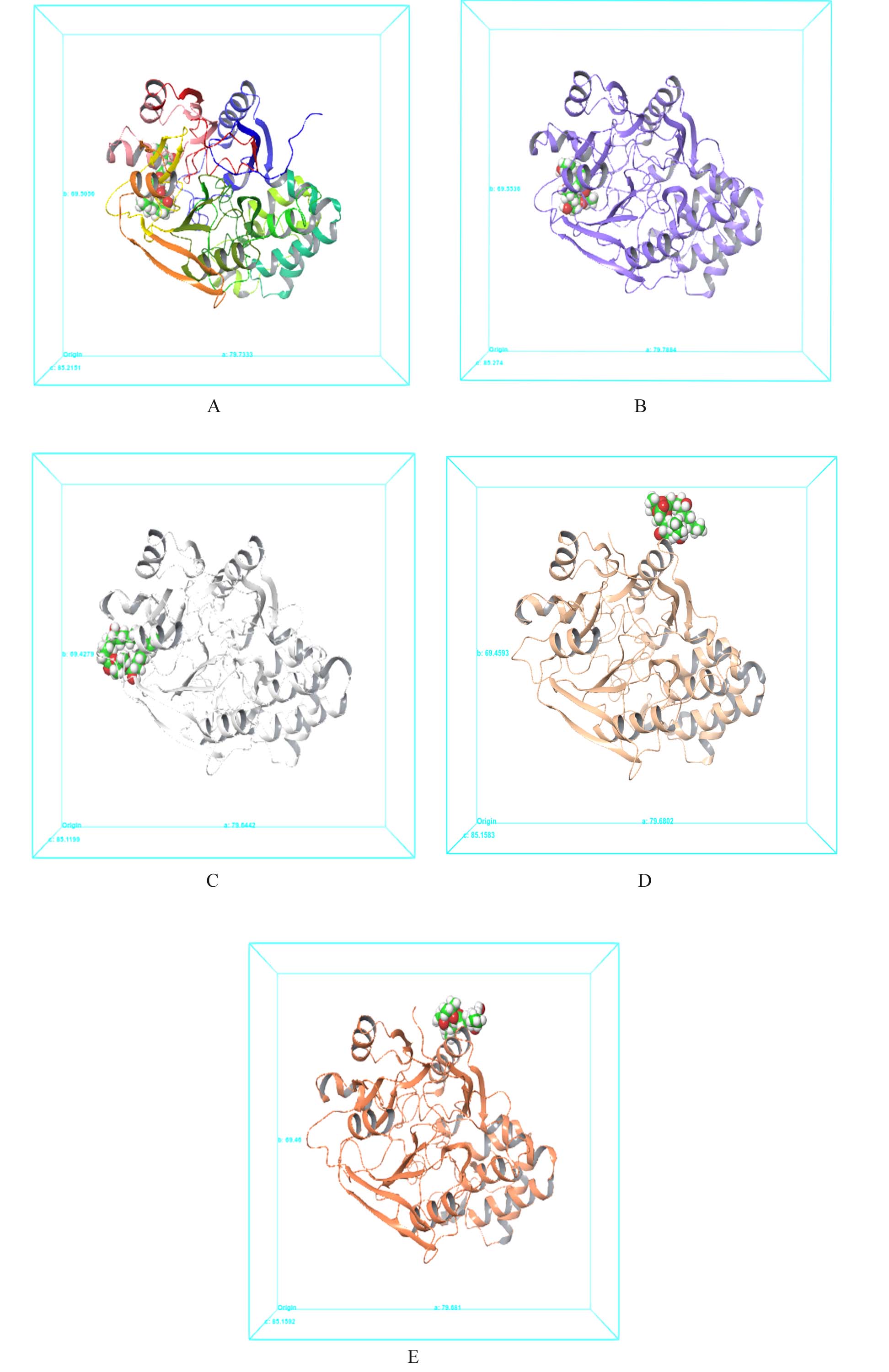


Supplementary Fig. 1. Protein ligand complex position at various time stages. Water molecules and ions have not been shown to visualize clear placement of the ligand. (A). First frame at 0 ns, showing ligand in CPK representation. Protein is shown in Ribbon (colored by secondary structure). (B). 1000^th^ frame at 16 ns, showing ligand in CPK representation. Protein is shown in Ribbon (colored by purple). (C). 3000^th^ frame at 48 ns, showing ligand in CPK representation. Protein is shown in Ribbon (colored by gray). (D). 4000^th^ frame at 64 ns, showing ligand in CPK representation. Protein is shown in Ribbon (colored by wheat). (E) 5000^th^ frame at 80 ns, showing ligand in CPK representation. Protein is shown in Ribbon (colored by orange).


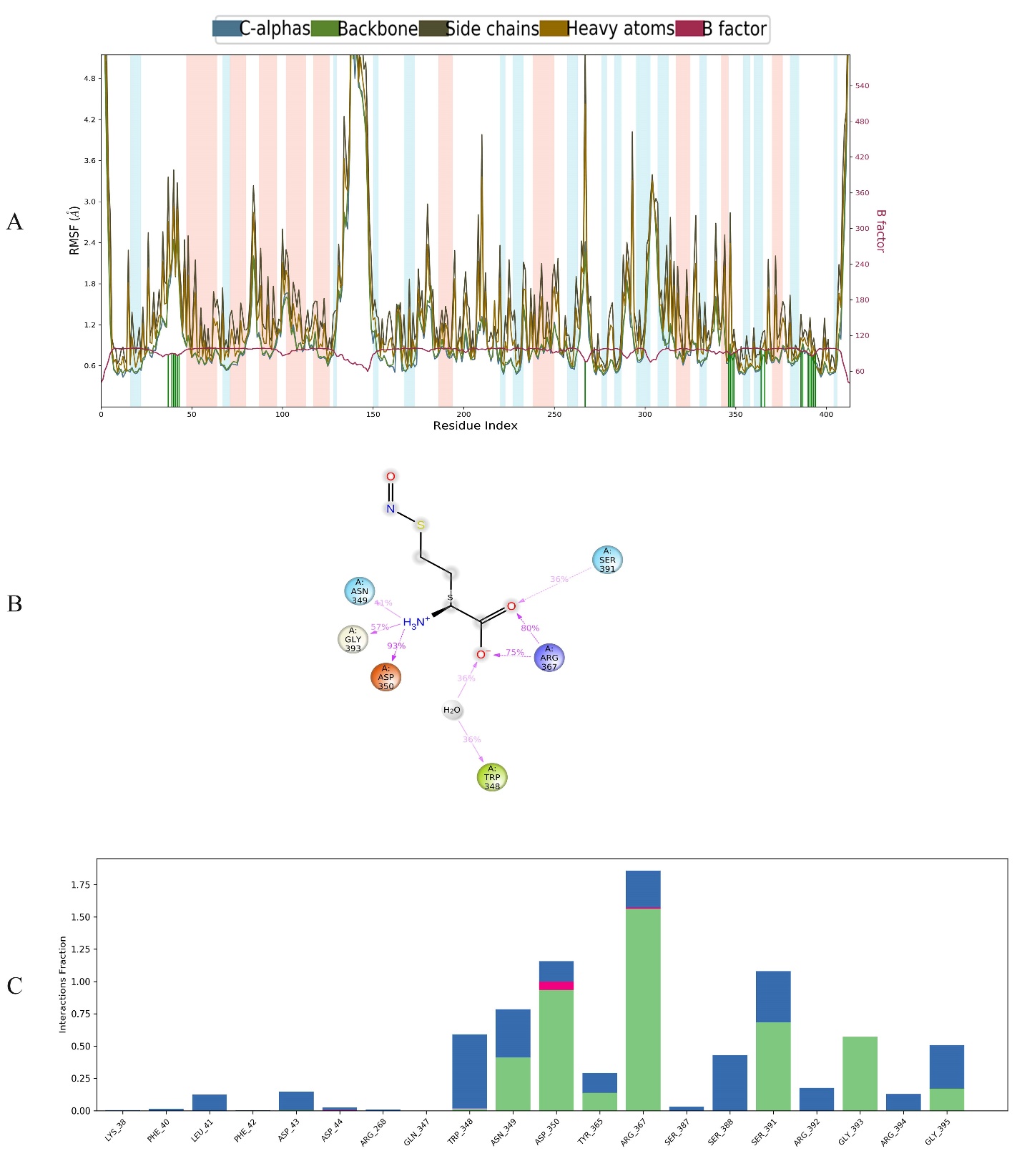


Supplementary Fig. 2. (A). RMSF of control compound (B)Residue interaction of control compound, retained for more than 30% of simulation time (C) Interaction fraction of residues during simulation. Hydrogen bonds shown in green, hydrophobic in mauve, ionic in pink and water bridges in blue.


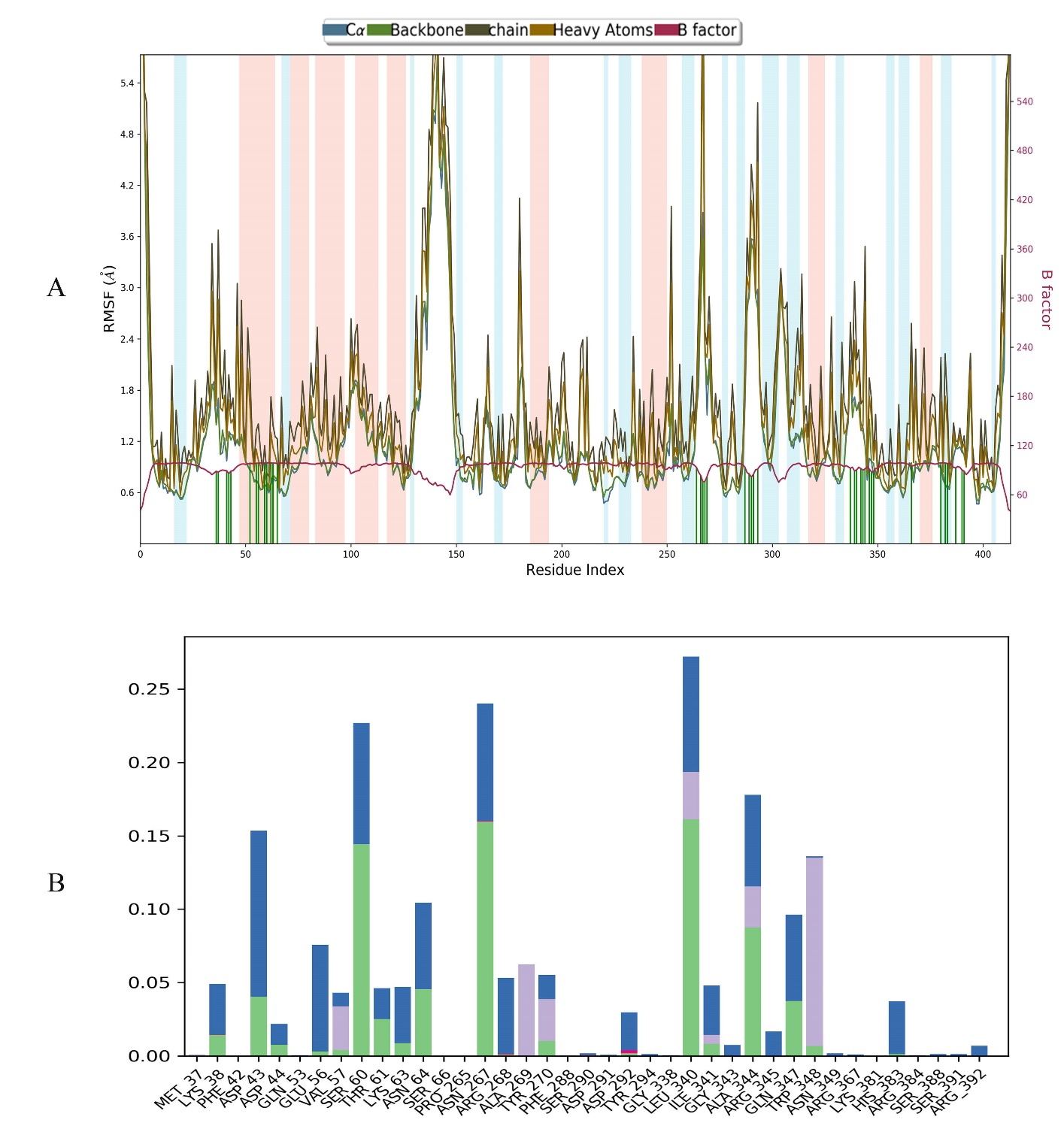


Supplementary Fig. 3. (A). RMSF of CMNPD18759 (B) Interaction fraction of residues during simulation. Hydrogen bonds shown in green, hydrophobic in mauve, ionic in pink and water bridges in blue.


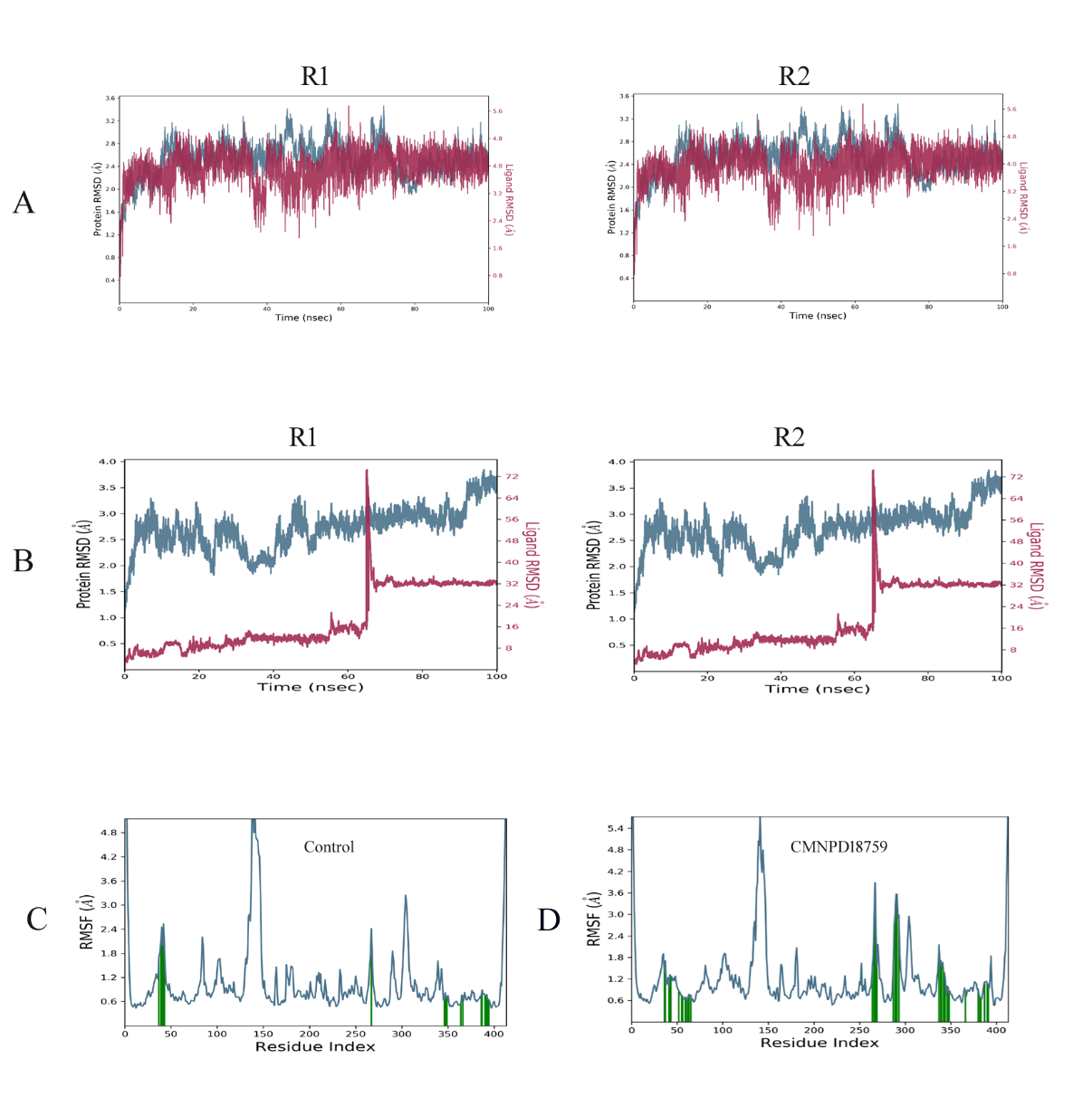


Supplementary Fig. 4. (A). RMSD plot of control and protein. R1 denotes first replicate and R2 denotes second replicate. (B). RMSD plot of CMNPD18759 and protein. R1 denotes first replicate and R2 denotes second replicate. (C) RMSF of control-protein complex. (D) RMSF of CMNPD18759-protein complex.
